# Directly imaging the localisation and photosensitization properties of the pan-mTOR inhibitor, AZD2014, in living cancer cells^☆^

**DOI:** 10.1016/j.jphotobiol.2020.112055

**Published:** 2020-12

**Authors:** Abdullah R. Ahmed, Alessia Candeo, Sofia D'Abrantes, Sarah R. Needham, Rahul B. Yadav, Stanley W. Botchway, Anthony W. Parker

**Affiliations:** a Central Laser Facility, Science & Technology Facilities Council, Rutherford Appleton Laboratory, Harwell Campus, Didcot, Oxfordshire OX11 0QX, UK; b Larch House, Woodlands Business Park, Breckland, Linford Wood, Milton Keynes MK14 6FG, UK; c CRUK/MRC Oxford Institute for Radiation Oncology, University of Oxford, Gray Laboratories, ORCRB Roosevelt Drive, Oxford OX3 7DQ, UK; d Evotec (UK) Ltd, 114 Innovation Drive, Milton Park, Abingdon, Oxfordshire OX14 4RZ, UK

**Keywords:** AZD2014, INK128, mTOR, Fluorescence microscopy, PDT, Photosensitizer, Multiphoton, GFP, FLIM;light sheet fluorescence microscopy, BSA, Bovine Serum Albumin, CHO, Chinese Hamster Ovary, ER, Endoplasmic Reticulum, FLIM, Fluorescence Lifetime Imaging, HEK293, Human Embryonic Kidney, LSFM, Light Sheet Fluorescence Microscopy, mTOR, Mechanistic Target of Rapamycin, MCF-7, Michigan Cancer Foundation-7, PBS, Phosphate Buffered Saline, PDT, Photodynamic Therapy, TCSPC, Time-Correlated Single Photon Counting

## Abstract

The range of cellular functions the mechanistic target of rapamycin (mTOR) protein performs makes it an attractive drug target for cancer therapy. However, the cellular localisation and mode of action of second generation inhibitors of mTOR is poorly understood despite the level of attention there is in targeting the mTOR protein. We have therefore studied the properties of the pan-mTOR inhibitor AZD2014, an ideal candidate to study because it is naturally fluorescent, characterising its photochemical properties in solution phase (DMSO, PBS and BSA) and within living cells, where it localises within both the nucleus and the cytoplasm but with different excited state lifetimes of 4.8 (+/− 0.5) and 3.9 (+/− 0.4) ns respectively. We measure the uptake of the inhibitor AZD2014 (7 μM) in monolayer HEK293 cells occurring with a half-life of 1 min but observe complex behaviour for 3D spheroids with the core of the spheroid showing a slower uptake and a slow biphasic behaviour at longer times. From a cellular perspective using fluorescence lifetime imaging microscopy AZD2014 was found to interact directly with GFP-tagged mTORC1 proteins including the downstream target, S6K1. We observe light sensitive behaviour of the cells containing AZD2014 which leads to cell death, in both monolayer and spheroids cells, demonstrating the potential of AZD2014 to act as a possible photodynamic drug under both single photon and multiphoton excitation and discuss its use as a photosensitizer. We also briefly characterise another pan-mTOR inhibitor, INK128.

## Highlights

• The mechanistic target of rapamycin (mTOR) is an attractive drug target for cancer therapy.
• Pan-mTOR inhibitors provide a means to target mTOR function to kill cells and treat disease.
• AZD2014 demonstrates potential to develop drugs that act both as a pan-mTOR inhibitor and photodynamic sensitizer.
• Multiphoton excitation excites the drug away from interfering light absorption by the cell.

## Graphical Abstract

**Figure:**
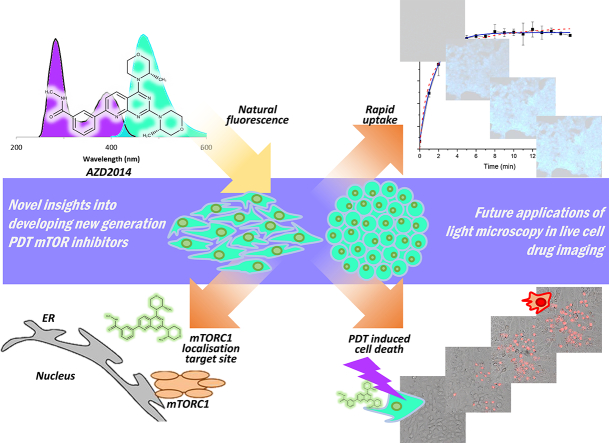

## 1 Introduction

**Fig. 1:**
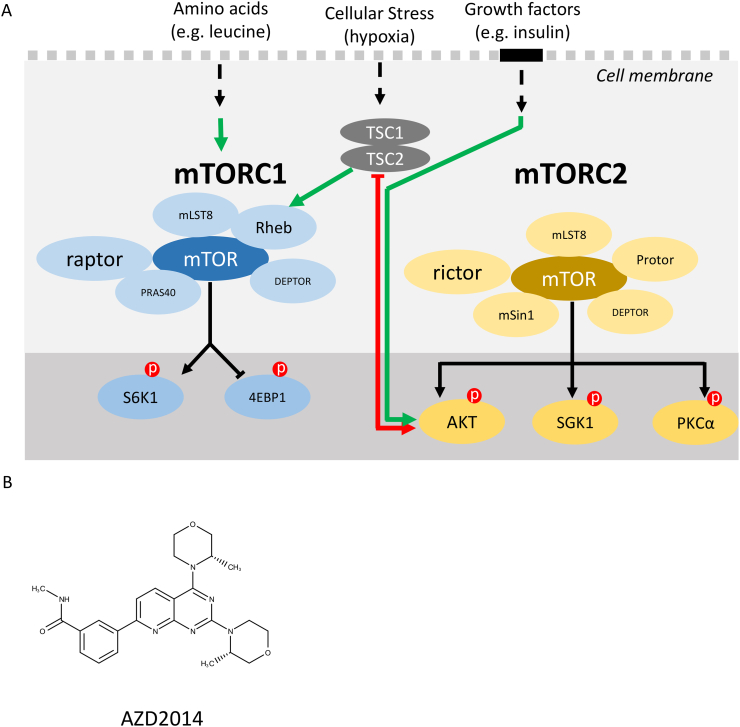
mTOR signalling in the cell at a glance. A) Blue filled circles show the mTORC1 protein and pathway, yellow filled circles show mTORC2 and its pathway. Green arrows show activation, red arrows show inhibition. (P) shows phosphorylation (activation). B) Chemical structure of AZD2014. (For interpretation of the references to colour in this figure legend, the reader is referred to the web version of this article.)

The mechanistic Target of Rapamycin (mTOR, earlier references also refer to m as mammalian) pathway has been established as being at the heart of numerous cellular signalling pathways, including small molecule sensing and responding to physiological and intricate biochemical changes within the cellular environment (Fig. 1A) [1]. The mTOR protein (290 kDa) is a serine/threonine kinase, belonging to the subgroup of phosphoinositide 3-kinase-related kinases (PIKKs), and functions to co-ordinate levels of nutrients, growth factors, oxygen, stress and energy (ATP) [2]. The mTOR signalling pathway is situated downstream of the well-known cell survival phosphoinositide 3-kinase (PI3K)/AKT pathway and can be linked to other signalling pathways [[3], [4], [5]].

Important roles of mTOR include its involvement in regulating cellular growth and proliferation [6], protein synthesis [7], autophagy [8], metabolism [2], aging [9], movement [10] and memory formation [11]. The mTOR pathway manifests its broad range of function by mTOR assembling into two multi-protein complexes, namely mTOR complex 1 (mTORC1) and mTOR complex 2 (mTORC2) which are structurally and functionally distinct [12]. Both complexes differ in their subcellular localisations which is imperative for mTORC1 phosphorylating (activating) its downstream target proteins 4EBP1 and S6K1 (protein synthesis), as well as for mTORC2 phosphorylating AKT (cell survival) [2].

It has been reported that mTORC1 is localised to subcellular structures and compartments within the cell, some of which include the endoplasmic reticulum (ER)/Golgi, mitochondria, lysosomes, peroxisomes, cytoplasm and nucleus [13,14]. Determining how the complex is assembled and identifying where it may localise is imperative for both advancing the understanding of the signalling pathway as well as for strategically targeting it.

The diverse functionality of mTOR provides a very attractive drug target where dysfunctional mTOR signalling can lead to diseases such as cancer, type II diabetes, obesity, aging and neurodegenerative conditions [12]. In particular, the last two decades have seen immense research efforts in drug design and development where inhibiting the mTOR kinase protein can prevent cancer growth [15,16]. It is reported that mTOR is hyperactivated in almost 70% of all human cancers [17]. Thus, the ability to manipulate the mTOR kinase through pharmaceutical intervention plays a fundamental part in today's battle against cancer.

The first generation inhibitors of mTOR (rapalogs) based on the rapamycin molecule, where mTOR's name is derived from, exhibited both anti-fungal and anti-proliferative properties when first discovered [18]. Accumulating evidence suggests a lack of efficacy of rapalogs as well as emerging rapalog resistance in a number of tumours [19]. The inefficiency of rapamycin and rapamycin analogues may be largely due to their mechanism of binding [20]. Rapamycin binds to an intracellular carrier protein FK506 Binding Protein-12 (FKBP12); this rapamycin-FKBP12 complex then binds to the FKBP12-rapamycin-binding (FRB) domain of mTOR [21]. It was originally thought that rapamycin caused the disassembly of mTORC1 [22,23], however, in light of recent structural studies and live cell studies using fluorescence lifetime imaging microscopy for protein-protein interactions [14], it appears this is not the case, but rather the binding of rapamycin-FKBP12 onto mTOR allosterically restricts the entry of mTOR substrates accessing the active site for mTOR mediated phosphorylation [24,25]. Rapamycin is reported to inhibit S6K1 completely and 4EBP1 incompletely on mTORC1 while its effects on mTORC2 appears to be uncertain and may be cell line dependant [26,27]. Thus, targeting the mTOR molecule by other means other than allosteric inhibition may provide greater potency.

Second generation kinase mTOR inhibitors, frequently referred to as mTORKis or TORKinibs, bind to the mTOR kinase domain, and are mechanistically ATP-competitive inhibitors that provide greater selectivity by inhibiting both mTORC1 and mTORC2 function [17]. These types of inhibitors have already been shown to be more effective than rapamycin and rapalogs [28]. Potential candidates that are currently undergoing clinical trials are the INK128 and AZD2014 molecules [29]. The AZD2014 molecule (Fig. 1B) has progressed greatly and was optimised from the successful AZD8055 compound. AZD2014 appears to be more soluble and less toxic (safer) for clinical use when compared to AZD8055. The AZD2014 molecule has an IC_50_ value of 0.0028 μM, making it one of the most effective inhibitors discovered and although it may bind to some PIKK proteins, it has been shown to be unselective towards a panel of 200 other tested kinases [30]. AZD2014 is promising as an anti-cancer reagent, particularly with combination therapies [31]. Preclinical [32], pharmacological characterisation [33] and xenograft models [34] in mice have been performed up to now. The exact mechanism of action of AZD2014 in live human cancer cells is yet unclear and warrants further investigation as studies have demonstrated that AZD2014 may bind directly to the kinase domain of mTOR [30] while other studies have shown mTOR complex dissociation with AZD2014 treatment [35].

Identifying where drugs localise within cancer cells and living tissues is imperative to both understanding the mechanism of pharmaceutical drugs and their effectiveness in binding to drug targets on a sub-cellular level [36,37] as well as to optimise their effect. Although most common ways to study drug localisation involve making fluorescent analogues of the drug [38,39], fixing/ablating samples as found in forms of mass spectroscopy imaging [40,41], stimulated Raman scattering microscopy has been mostly relied upon to directly image drug localisation in living cells [42]. However, the use of natural fluorescence as a direct tool for live cell drug imaging has been largely overlooked and we seek to address this fundamental aspect of AZD2014.

This study aims to characterise the intrinsic fluorescent nature of the second generation mTOR inhibitor, AZD2014, in order to provide information on its spectral properties that would be used for the application of multimodal live cell imaging of the inhibitor in real-time. The ability to use fluorescence-based imaging techniques such as confocal and multiphoton microscopy, Fluorescence Lifetime Imaging Microscopy (FLIM) and Light Sheet Fluorescence Microscopy (LSFM) represents a very powerful, insightful and novel avenue for drug target investigation and development, for both mTOR pathway studies and its associated protein-protein and protein-drug interactions and cancer therapy [43,44]. We have previously shown that FLIM is a powerful tool in understanding both the molecular environment and protein-protein interactions in live cells [14,45]. We identify firstly the AZD2014 subcellular localisation in live human cancer cell adherent models (HEK293, MCF-7 and CHO), secondly we characterised its uptake from the same monolayers as well as from 3D multicellular spheroids (HEK293). Thirdly, we investigated the interactions of AZD2014 with the mTORC1 proteins including the downstream target, S6K1. In general, where and how mTOR inhibitors localise is imperative and crucial in drug design, development and towards their mechanism of action. Furthermore, we explore the interaction of AZD2014 with proteins in the mTORC1 unit, the mechanism of the drug in relation to both light activation and cell death, showing photodynamic activity.

Our study is driven by the need for a comprehensive understanding of newer second generation mTOR inhibitors, such as AZD2014, seeking to optimise more effective delivery into specific cellular domains that will ultimately lead to better therapies. These results demonstrate the potential of how advanced imaging methodologies provide a means to merge drug discovery and development. The experimental methods are summarised in Table 1.

**Table 1:** Summary of spectroscopic and imaging experimental techniques employed.

Method of investigation	Parameters		Experiment/samples
	Excitation	Emission	
Multiphoton including FLIM	600 and 800 nm	460 ± 60 nm and BG39 filter	AZD2014 /GFP, monolayer cells
Confocal microscopy	400/488/561 nm	460 ± 60 nm/525 ± 39 nm/ 633 IU-25 filter	Observed uptake and localization of AZD2014 in 2D monolayer cells as well as PDT effect ADZ2014/GFP/mCherry
Light sheet microscopy	400 nm		Observed uptake and PDT effect of AZD2014 in 3D spheroids and monolayers
Spectroscopy (UV–Vis, Fluorimeter)	UV–vis to 800 nm	BG39 for multiphoton	Characterisation of absorption, emission and quantum yields for monolayer cells and solution phase

## 2 Materials and methods

### 2.1 Materials

All chemicals and reagents in this work were purchased and used without further treatment or purification. General laboratory reagents were purchased from Sigma-Aldrich (UK) unless otherwise stated. AZD2014 and INK128 were purchased from MedChem Express (Europe). Cell culture dishes (35 mm × 20 mm) were bought from MatTek (USA) and 96-well round plates from (Corning™ Falcon™). Propidium iodide was purchased from Sigma-Aldrich (UK). ER-Tracker™ Red and LysoTracker Red DND-99 was purchased from Thermofisher Scientific (UK). mDsRed-Rheb, EGFP-Rheb, EGFP-mTOR and EGFP-S6K1 DNA plasmids made as previously described [14] [45]. EGFP-raptor plasmid was a kind gift from Prof. Jacek Jaworski (Poland) and purified GFP was a kind gift from Prof. Cameron Naylon (Australia).

All cell culture reagents were purchased from Thermofisher Scientific (Gibco™).

Human Embryonic Kidney (HEK293) cells were purchased from ATCC (U.S.A.), certified as mycoplasma (contamination) free. Michigan Cancer Foundation-7 (MCF-7) breast cancer and Chinese Hamster Ovary (CHO) cells were initially purchased from ECACC (UK) and mycoplasma free.

### 2.2 Cell culture

HEK293 cells were cultured in phenol-red free Minimum Essential Media (MEM) supplemented with 10% Foetal Bovine Serum (FBS), 2 mM l-glutamine (L-glut) and 1% Penicillin-Streptomycin (Pen-Strep) for HEK293. MCF-7 and CHO cells were cultured in DMEM supplemented with 10% FBS, 2 mM L-glut and 1% Pen-Strep. Cells were grown in 5% CO_2_ at 37 °C, passaged three times a week once ~80% confluency had been reached and seeded at a density of 1.2–1.5 × 10^5^ cells/ml in 35 mm glass bottom dishes.

### 2.3 Trypan blue exclusion test of cell viability

To accurately determine cell viability with AZD2014 treatment, varying concentrations of AZD2014 (0–20 μM) were added to trypsinised HEK293 cells for 30 min. 10 μl of treated cells were mixed with 10 μl of trypan blue (Bio-Rad) in a 1:1 ratio, loaded into a counting slide chamber and number of live cells determined by using a TC20 automated cell counter (Bio-Rad).

### 2.4 Transfecting mammalian cells with plasmid DNA

Following 24 h after cell seeding, 500 ng of fluorescently tagged mTORC1 plasmid DNA was mixed with 6 μl of FuGENE HD (Promega) and the rest of the transfection complex made to 100 μl in Opti-MEM™ Reduced Serum Media (Gibco) in a total volume of 1 ml. DNA: transfection complex was vortexed briefly and incubated for 10 min at room temperature before being added dropwise to the media of seeded cells.

### 2.5 Mammalian cells spheroid culture

HEK293 cells were seeded into 96-well round (U-well) bottom plate at a cell density of 10,000 cells per well with a final volume of 200 μl in complete growth media. Each well was pre-cast with 100 μl of 1.5% low gelling temperature agarose (Sigma-Aldrich) in water prior to seeding. 200 μl of PBS (1×) was added to the outer wells of the 96-well plate to maintain humidity due to evaporation losses from inner wells (Supplementary Fig. S.1A). The plate was incubated in 5% CO_2_ at 37 °C for 3–4 days until spheroids were formed in suspension above the agar layer. The spheroids were then transferred to low gelling point agarose holders, in which groves obtained with a 3D printed comb were cast, hosting up to 5 spheroids (Supplementary Fig. S.1B-D).

### 2.6 Colocalisation cellular and cell death studies

Adherent HEK293 cells grown to 70–80% confluency were incubated with 7 μM AZD2014 and subsequently stained with ER-Tracker™ Red and LysoTracker Red DND-99, per manufacture's protocol.

HEK293 monolayer or spheroid cells were co-stained with AZD2014 (7 μM) and propidium iodide (1.2 μg) by diluting the compounds into 0.5 ml of complete growth media and then administrating it drop-wise to adherent cells (in dishes) in a final media volume of 2 ml or to spheroids mounted for imaging with 5 ml of final media volume.

### 2.7 UV–vis and fluorescence studies

Inhibitor concentrations of rapamycin (10 μM), AZD2014 (7 μM or 11 μM) and INK128 (7 μM or 16 μM) were prepared in DMSO (Thermofisher Scientific) and diluted in other solvents such as 0.5–1 mM bovine serum albumin (BSA), water or phosphate-buffered saline (PBS) by vortexing and stored at −20 °C in the dark. GFP solutions were also prepared in deionised water. UV–vis spectra were taken using a Shimadzu UV-1800 spectrometer and fluorescence measurements using an Agilent Technologies Cary Eclipse Fluorescence Spectrophotometer. The fluorescence quantum yields (QY_f_) were determined using well established standards, Coumarin-1 (Exciton, UK), Quinine (Sigma-Aldrich) in 0.5 M sulphuric acid and Rhodamine 6G (Exciton, UK) using Eq. (1) [46].

(1) $$QY_{f}=Q_{R}\frac{I}{I_{R}}\frac{{\mathrm{OD}}_{R}}{\mathrm{OD}}\frac{n^{2}}{n_{R}^{2}}$$

where QY_f_ is the unknown quantum yield of the sample, *Q*_*R*_ is the quantum yield of the standard, *I* is the integrated fluorescence intensity of the sample, *I*_*R*_ is the integrated fluorescence intensity of the standard, OD_R_ is the optical density of the standard, *OD* is the optical density of the sample, *n*^*2*^ is the refractive index of the sample and *n*^*2*^_*R*_ is the refractive index of the standard.

The multiphoton excitation of AZD2014 was obtained using the Leica TCS SP8 in xyλ acquisition mode, which allows one to record a series of images at different excitation wavelengths (from 680 to 1300 nm) with an InSight® DS ultrafast (120 fs pulse width) laser system (Spectra-Physics, UK), stepping the excitation wavelength of 5 nm at a time.

### 2.8 One-photon and multiphoton flim imaging of AZD2014 and INK128

Confocal images were taken using an inverted Nikon TE2000-U or Ti-E microscope attached to a Nikon C1 or C2 scanning unit with 405, 543 or 561 nm excitation and appropriate filter set or using a Leica TCS SP8X confocal microscope using internal pre-set BFP, GFP, mCherry software settings and filters or using a Zeiss LSM 880 with Airyscan using pre-set BFP filter settings. For multiphoton excitation and FLIM, the system has been reported previously [47]. Two photon studies were undertaken using 600 ± 5 nm wavelength from a Coherent APE ring cavity optical parametric oscillator.

### 2.9 Uptake studies in cells and spheroids

For uptake in cells, confocal images over a 20 min time period with one minute time intervals were acquired using 405 nm excitation. The fluorescence intensities of the compounds over time were extracted using ImageJ and then fitted using the Michaelis–Menten function [48].

Following 72 h of seeding in agarose-coated 96 U-well plates, HEK293 cell spheroids were transferred into the agarose holder in a 35 mm glass bottom dish and filled with complete growth media. The samples were imaged at 37 °C and 5% CO_2_. The Leica TCS SP8 DLS (Digital Light-Sheet, Supplementary Fig. S.1E,F) was used to acquire 3D time-lapse images of spheroids after administration of AZD2014. The compound AZD2014 was administrated at a final dose of 7 μM in the media, and uptake into spheroids was monitored for 2 h by recording stacks of 780 μm × 780 μm × 300 μm volume composed of 44 planes taken every 15 s. Time series stacks of images were acquired using a 10×/0.3 NA detection objective, with a field of view of 735 μm × 735 μm. For the light sheet generation, laser light at 405 nm wavelength was used, focused with a 2.5×/0.07 NA objective and scanned by galvanometric mirrors, digitally creating a light sheet with a thickness of 3.7 μm and a Rayleigh length of 240 μm. Two counter-propagating light sheets were also used in order to reduce blur of the image due to scattering of light throughout the sample as well as striping effects. Each frame was acquired with one light sheet at a time and merged into a single image. The laser power at the sample was 200 μW, distributed in the light sheet.

### 2.10 Data analysis software

TCSPC and FLIM data analysis was performed in SPCImage version 6.0. Generally the data fitted to a single exponential and gave an extremely good fit to single exponential (characterised by a Chi-square of 1) and fitting to a double exponential was deemed inappropriate. Extracting and analysing confocal and Light sheet images was performed in ImageJ (Fiji) [49].

## 3 Results

### 3.1 Fluorescence properties of AZD2014

**Fig. 2:**
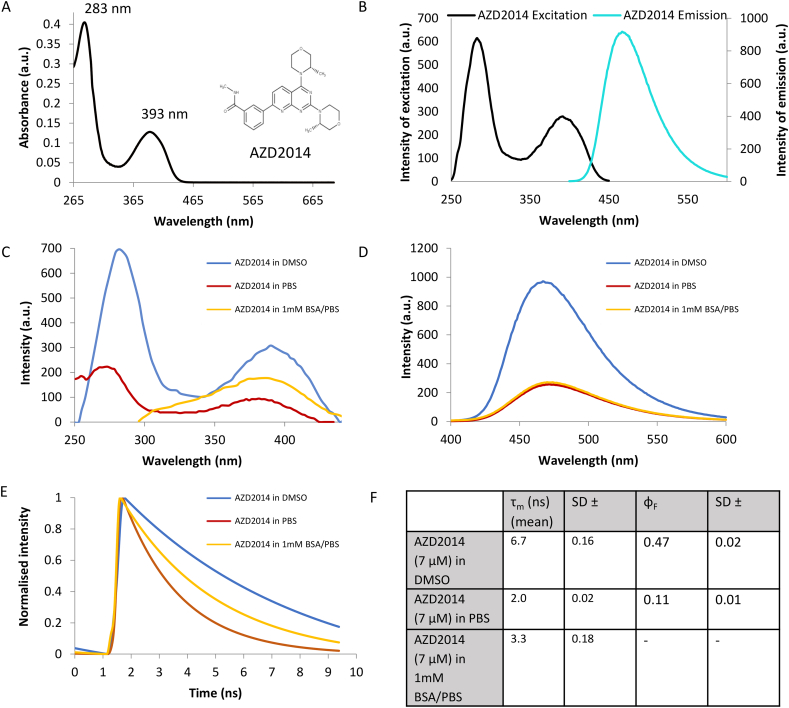
Fluorescence characterisation of AZD2014. A) UV-VIS spectrum of AZD2014 (11 μM) in DMSO solvent. B) Fluorescence spectrum of AZD2014 (7 μM) in DMSO showing excitation profile in black and emission profile in cyan. Spectra shown on two different axis and were taken at different detector settings to provide clean spectrum. C) Excitation spectra of AZD2014 in various solvents (DMSO, PBS and 1 mM BSA/PBS). D) Emission spectra of AZD2014 in various solvents (DMSO, PBS and 1 mM BSA/PBS). E) Fluorescence intensity decays of AZD2014 in various solvents (DMSO, PBS and 1 mM BSA/PBS). F) Summary of lifetimes and quantum yields. Data repeated minimum of three independent times. (For interpretation of the references to colour in this figure legend, the reader is referred to the web version of this article.)

The solvent corrected UV–Visible absorption spectrum of AZD2014, Fig. 2A, shows two absorbance maxima 283 and 393 nm with molar extinction coefficients (ε) 36,790 and 11,660 M^−1^ cm^−1^ respectively (see Supplementary Fig. S.2A). We identify the optimum excitation wavelength for cell studies as 393 nm based on the extinction coefficient of EGFP 55,000 M^−1^ cm^−1^ at 488 nm lowering to 8600 M^−1^ cm^−1^ at 393 nm [56].

The fluorescence emission spectra of AZD2014 (7 μM) in DMSO is given in Fig. 2B, showing an emission maximum at 468 nm. The emission maximum shows some solvent dependence with the emission maximum shifting as demonstrated. To identify any solvent dependence of AZD2014 fluorescence, other solvents were also investigated, PBS and BSA (1 mM) diluted in PBS, the latter representing a serum protein mimicking a cytosol and nucleosol protein environment of the cell. Fig. 2C, we observe a 7 nm shift in the excitation spectrum of AZD2014 in PBS and BSA compared to DMSO as well as a notable decrease in the absorption intensities. Fig. 2D shows the relative fluorescence intensities of AZD2014 is quenched in both PBS and BSA solutions (by 72%) compared to DMSO indicating that the solvent environment has a significant effect on the spectral emission properties of AZD2014. These differences were further mirrored in the fluorescence lifetime of the molecule in these solutions extracted from raw intensity decays (Fig. 2E-F) measured using Time-correlated Single Photon Counting (TCSPC) with AZD2014 showing a single exponential lifetime of 6.7 ± 0.161 ns in DMSO, and a shorter single exponential lifetime of 2.0 ± 0.023 ns in PBS. On the other hand AZD2014 in BSA gave a double exponential decay, with lifetimes 0.97 +/− 0.29 and 5.16 ±0.71 ns, giving a weighted average lifetime of 3.25 +/− 0.18 ns. In both DMSO and PBS solutions the optical densities (OD) of AZD2014 were similar (spectra in Supplementary Fig. S.2B) indicating a solvent dependant fluorescence quantum yield. The florescence quantum yield (QY_f_) of AZD2014 was determined using two fluorescent standards, coumarin-1 (QY_f_ of 0.73 in ethanol) [44] and quinine sulphate (QY_f_ of 0.55 in sulphuric acid) [45] (see Materials and methods for details). Using 393 nm excitation we obtain AZD2014 QY_f_ values for DMSO = 0.47 ± 0.02, PBS 0.11 ± 0.01 (see Supplementary Fig. S.2). The 74% decrease in quantum yield observed in PBS supports the decrease in AZD2014 fluorescence emission and indicates AZD2014 may be brighter in environments similar to DMSO than PBS. Fig. 2F gives a tabulated summary of the fluorescence data obtained.

Next we sought to characterise the two-photon excitation absorption characteristics of AZD2014 using two excitation wavelengths, 798 nm and 680 nm (Supplementary Fig. S.2B).

### 3.2 Uptake, localisation and quantification of AZD2014 in monolayer cells

**Fig. 3:**
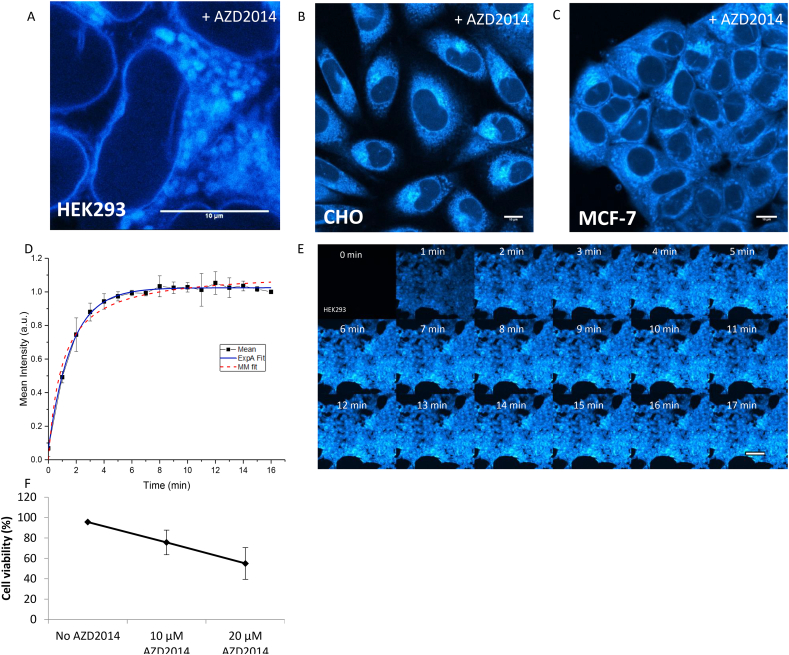
Live cell uptake and imaging of AZD2014. Confocal imaging of AZD2014 (7 μM) in live A) HEK293 cells, B) CHO cells and C) MCF-7 cells using 405 nm excitation. Scale bar = 10 μm. D) Plot of extracted confocal intensities of AZD2014 (7 μM) over the 17 min with Michaelis–Menten (MM) and non-linear (ExpA) fitting. E) Confocal images of AZD2014 (7 μM) uptake in HEK293 cells taken over 17 min of administration, with one minute time intervals using 405 nm excitation. Scale bar = 50 μm. F) Trypan blue exclusion test of HEK293 cell viability with AZD2014 treatment. Repeated minimum of three independent times. Error bars show standard deviation from three experiments. (For interpretation of the references to colour in this figure legend, the reader is referred to the web version of this article.)

Following on from the solution studies, imaging the uptake of AZD2014 in live mammalian cells was investigated. A solution of AZD2014 (7 μM) in complete growth media was added to adherent exponentially growing HEK293 cells. Fig. 3 shows that AZD2014 was readily taken up by HEK293 cells within 1 min of addition (k_uptake_ = 1 min, defined by time at 1/2 the maximum intensity saturation) and plateaued at around 4 min. After 15 min the uptaken AZD2014 in HEK293 cells was imaged by fluorescence confocal microscopy and showed localisation in the nucleus, the cytosol and peri-nuclear subcellular regions of the cell (Fig. 3A). Both, CHO cells and MCF-7 cells showed a similar localisation pattern as HEK293 cells, see Fig. 3B and Fig. 3C. Cell viability was tested at different AZD2014 concentrations using the trypan blue exclusion test. At concentrations similar to that administered in the cell studies (10 μM treatment of AZD2014 at 30 min, Fig. 3F), the test shows at least 80% cell viability. It is worth noting the 7 μM AZD2014 is similar to the concentration currently used in pre-clinical studies of 10 μM [50].

**Fig. 4:**
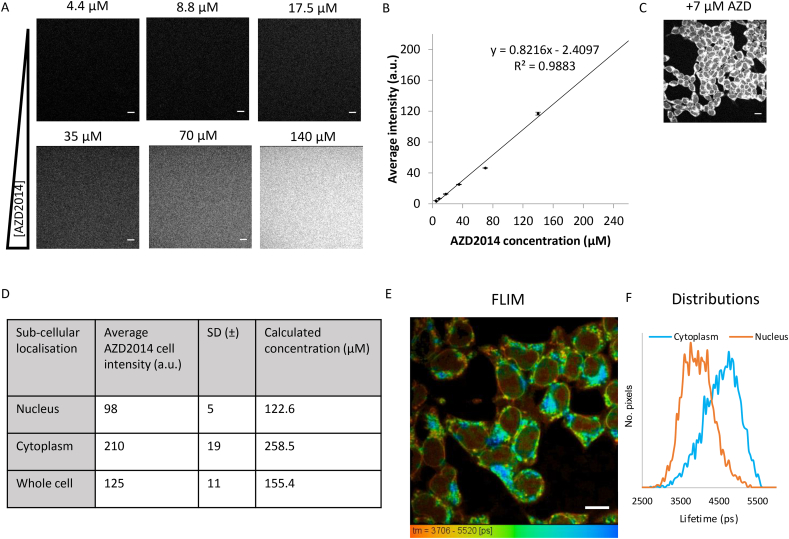
Quantification of AZD2014 in live HEK293 cells. A) Two-photon confocal images of AZD2014 in complete growth media (without cells) using 600 nm excitation at 1 mW. B) Calibration line graph of increasing concentrations of AZD2014 in complete growth media (without cells) with extracted intensities using 600 nm excitation. C) Two-photon confocal image of HEK293 cells treated with 7 μM AZD2014. D) Extrapolated nuclear, cytoplasmic and whole cell concentrations in HEK293 cells administered with 7 μM AZD2014. E) FLIM of AZD2014 in HEK293 cells with respected lifetime distributions, colours represent lifetime and not intensity vaues given in Fig. 4D. F) Normalised lifetime distribution plots for cytoplasm and nucleus (*n* = 9). Error bars show standard deviation. Scale bar = 10 μm, *n* > 3.

The concentrations of AZD2014 in living cells was determined next by measuring the average fluorescence intensity of various concentrations of AZD2014 in media solution with 600 nm two-photon excitation to construct a concentration calibration graph, see Fig. 4. Two-photon excitation was chosen over single (405 nm) excitation because of the advantage of better selective excitation and reduced cell photo-damage, induced by 405 nm absorption by other cellular components [51]. The 600 nm wavelength was chosen as the optimum laser wavelength that matches closest to 2 × 283 nm excitation of AZD2014 and easily achievable with our system. Under the same acquisition settings, the fluorescence images of HEK293 cells treated with 7 μM of AZD2014 were acquired 15 min following administration to allow the uptake to plateau. The drug concentrations in different locations of the cells were then extrapolated from the calibration graph. The accumulated concentration of AZD2014 in the nucleus was estimated as 124 μM and in the cytoplasm as 213 μM whilst the average concentration in the whole cell was determined as 148 μM (Fig. 4D). These findings suggest that the initial dosage administered (7 μM) does not relate to the final concentration obtained within the cell.

To obtain further information about the cellular environment where the drug accumulates, FLIM was employed. HEK293 cells administered with AZD2014 were imaged after 15 min and single exponential decays were observed with a longer lifetime of 4.8 ± 0.5 ns in the cytoplasm compared to the nucleus (3.9 ± 0.4 ns) as shown in Fig. 4E-F, the 1 ns difference in lifetime is significant, with the longer lifetimes indicating a lipid rich endomembrane environment such as ER/Golgi and perinuclear. It is worth noting that mTOR is known to reside in similar membrane bound organelles [13].

HEK293 cells administered with AZD2014 (7 μM) were co-stained with ER-Tracker Red to verify the specific site of localisation. As shown in Supplementary Fig. S.3, both AZD2014 and ER-Tracker Red showed strong co-localisation in living cells indicating that AZD2014 localises similarly to the ER, supporting the notion that both AZD2014 and mTORC1 are bound together in endosomal membranes. In addition, further colocalisation was carried out using Lyso-tracker (Supplementary Fig. S.3) and poor co-localisation was identified, although this may be due to the dynamics and cell nutrient dependency of mTORC1 within the live cell.

The co-localisation of AZD2014 with the Rheb subunit of mTORC1 was also explored due to its similar localisation and its function to tether mTORC1 to endosomal membranes such as the ER and Golgi [14]. AZD2014 (7 μM) was administered to HEK293 cells transiently overexpressing mDsRed-Rheb. Steady-state co-localisation analysis using the co-localisation colour map plugin for ImageJ [52], between AZD2014 and fluorescently labelled Rheb showed mapped regions of co-localisation with strong yellow-red ‘hot-spot’ areas indicating strong co-localisation particularly in the ER/Golgi sub-cellular regions of the cell, see Supplementary Fig. S.3H. The index of correlation (Icorr) value which represents the fraction of positively correlated pixels in the image was determined as 0.546, indicating that AZD2014 may localise to specific subcellular regions where active Rheb (known to be physically attached to mTOR) localisation sits (*i.e.* ER/Golgi membranes) [14].

The possible use of the fluorescence properties of AZD2014 for FRET was also explored. Attempts to use AZD2014 as a donor molecule and GFP (tagged to the mTORC1 proteins) as an acceptor molecule for FRET-FLIM studies were performed for piloting direct drug-protein interactions in living cells. An overlap (73%) of EGFP absorbance with the emission of AZD2014, in solution, (Supplementary Fig. S.4A) was determined. The fluorescence lifetime of a drop of AZD2014 in ethyl glycerol alone was measured as 4.0 ± 0.22 ns using 600 nm excitation since 405 nm excitation was found to be unsuitable due to simultaneous excitation of EGFP (data not shown). However, using two-photon excitation (600 nm) (200 fs, 76 MHz) and monitoring emission at 460 ± 60 nm, GFP emission and/or cellular autofluorescence is eliminated (Supplementary Fig. S.4C). A 1:1 ratio of AZD2014 and purified GFP was made in ethyl glycerol and gave a shortened AZD2014 lifetime of 2.70 ± 0.24 ns, see Supplementary Fig. S.4B, indicating direct physical interaction. When taken into the cellular model of HEK293 cells expressing EGFP-S6K1, EGFP-mTOR, EGFP-raptor and EGFP-Rheb proteins with AZD2014 (35 μM) treatment, changes in the natural lifetime of AZD2014 alone was found to show FRET efficiency between 17 and 27% using 600 nm excitation (Supplementary Fig. S.4C-E), suggesting the molecule directly interacts with mTORC1. A higher concentration (35 μM) was used for the interaction studies compared to uptake studies described above (7 μM). The higher concentration was necessary to ensure good signal-to-noise levels.

### 3.3 Uptake of AZD2014 into multi-layered spheroids

**Fig. 5:**
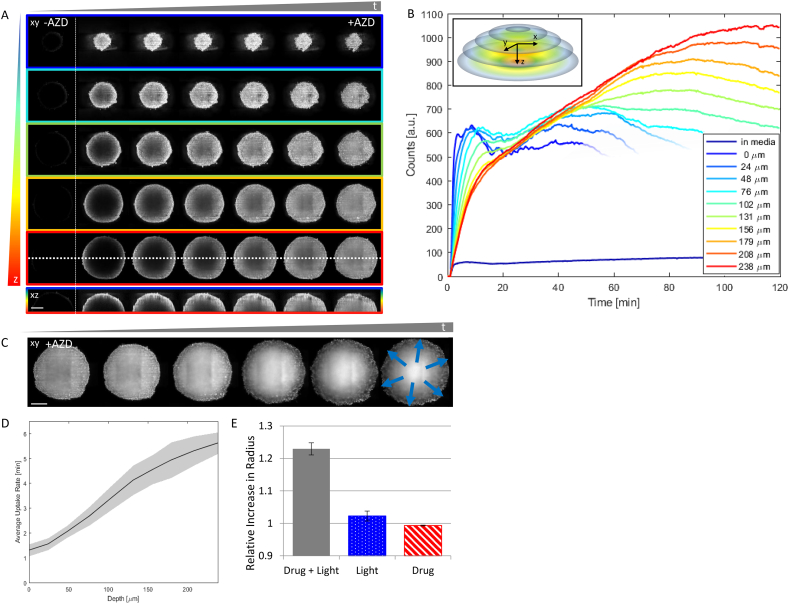
Uptake of AZD2014 in 3D multi-layered spheroids. A) AZD2014 (7 μM) administration to HEK293 spheroids imaged using light sheet fluorescence microscopy for 30 min. Images of planes acquired at different xy planes or depths (rows) and at different delays after AZD2014 administration (columns). The bottom row shows the orthogonal projection (xz plane) along the dotted line in the previous row. Images with/without AZD2014 treatment are shown (+/−). Scale bar 150 μm. B) Uptake of AZD2014 fluorescence in the spheroid over 2 h was studied at different depths from the surface. The inset shows the colour coding used for the curves extraction. C) Image planes at 250 μm in depth at different time points from 30 min to 2 h after administration. The blue arrows indicate the direction of increase in spheroid radius. Scale bar 150 μm. D) Mean rates of uptake, determined as the time at half-saturation of the fluorescence, for the various depths are shown. E) Graph showing relative increase of spheroid radius in comparison with the radius at time zero with AZD2014 only (red diagonal fill), 405 nm light only (blue dotted fill) and both AZD2014 and 405 nm (solid gray fill) treatments. Error bars show SD, n > 3. (For interpretation of the references to colour in this figure legend, the reader is referred to the web version of this article.)

To help assess the drug efficacy in a tissue environment, we characterise the uptake of AZD2014 in a spheroid tumour model mimicking the three-dimensional physiology and functions of living tissue. HEK293 spheroids were formed as described in Materials and methods and studied by means of LSFM. The AZD2014 fluorescence increased rapidly over time in the entire spheroid, with the outer layers showing a faster rate of uptake than the core of the spheroid (Fig. 5) as evident by the analysis of uptake rates at three selected depths of 0 (surface), 100 μm and 200 μm that gave values of 80, 200 and 318 s. Fig. 5B shows that the uptake curve is different in profile at different radial depths z, and also different from the behaviour predicted by the Michalis-Menten uptake model. The mean rates of uptake, determined as the time at half-saturation of the fluorescence, for various depths are shown in (Fig. 5D) and display a non-linear behaviour. The outer layer displays an initial high accumulation of the drug, with a mean uptake rate similar to the one recorded for monolayers. However, a reduction in the fluorescence was observed at longer time-points, which could be attributed to an exchange between the surface cells and those of the core. This latter point also shows a slow biphasic behaviour in the fluorescence time-course. It is worth noting that at the outermost layers (<50 μm) the uptake analysis beyond 60 min is not considered due to possible photobleaching from excessive excitation. Furthermore due to the photosensitisation, see below, it is assumed that these cells may be inactivated and this is expected to influence the uptake rates.

After 30 min following the administration of AZD2014 (7 μm), a 23% (std = ±0.019) increase in radius of the spheroid portion that was illuminated was observed (Fig. 5C and Fig. 5E). The cells of the spheroid appeared to swell and possibly undergo cell death, observable also from transmitted light imaging (data not shown). Further accumulation of drug into the spheroid core takes place, as seen by an increase in fluorescence intensity, most likely due to disruption in cell-cell contacts that ‘loosen’ the spheroid. Control experiments were also performed with 405 nm illumination only or AZD2014 (7 μM) only. The first control experiment showed an increase of 2.3% (std = ±0.02) in spheroid radius, while the second one did not give any relevant radius variation during the observation time, (Fig. 5E), supporting a photo-activated effect of AZD2014 (Fig. 5). Since cell swelling is a known marker for cell death [53], these results may implicate AZD2014 as a potential photosensitizer agent towards combined mTOR targeting and photodynamic therapy (PDT) against tumours and this aspect was investigated further, see below.

### 3.4 Cell death induced by photo-activation of AZD2014 in monolayer cells

**Fig. 6:**
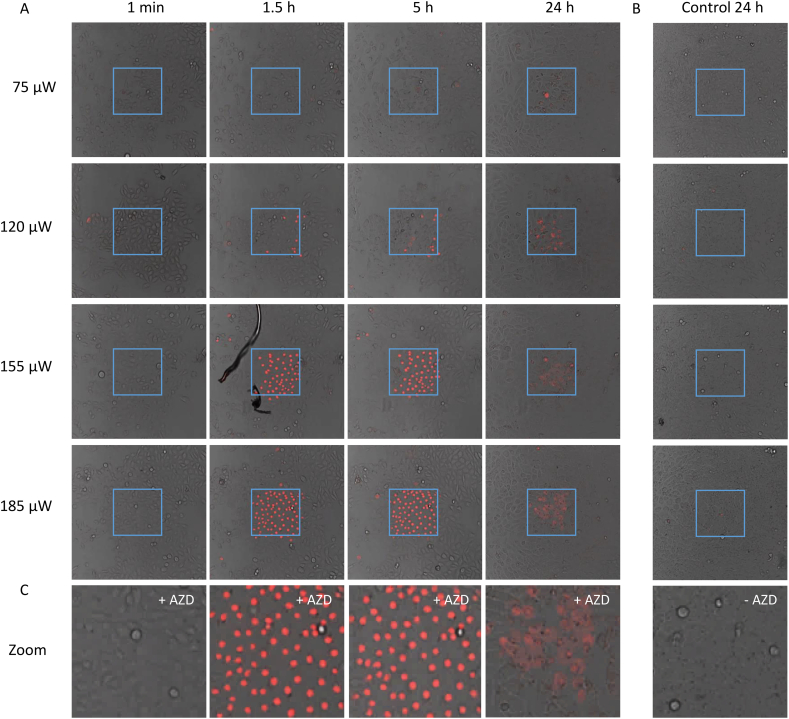
PDT of AZD2014 in adherent monolayer cells. A) Compilation of confocal overlay channel (561 nm and transmitted light) images over time and laser power showing cell death (as indicated by red fluorescence staining of PI) in CHO cells following treatment of AZD2014 (7 μM) and then 405 nm (CW) irradiation of FOV shown in cyan blue box (n > 3). B) Images of irradiated CHO cells without drug treatment but irradiated in cyan blue box (n > 3). C) Zoom of control and photoactivated cell death. (For interpretation of the references to colour in this figure legend, the reader is referred to the web version of this article.)

In order to verify the photosensitization properties of AZD2014, CHO cell monolayers were incubated with propidium iodide (PI) (to assay cell death) and 7 μM of AZD2014 for 20 min. A region of interest of the field of view was irradiated with the 405 nm laser at different powers for 10 min, and was monitored over 24 h (Fig. 6). The combined incubation and laser illumination led to positive PI staining in the exposed area as evident by the presence of fluorescence. A laser power of 75 μW (at the sample) resulted in cells detaching from the central area 24 h following irradiation. PI localization in the nuclei was observed 1.5 h after irradiation at a power of >150 μW in most of the cells. The rest of the field of view, which was treated with AZD2014 but not irradiated, remained unaffected. At a concentration of 3.5 μM, the required laser power to observe cell death was 185 μW or higher (Supplementary Fig. S.5). Control samples in which no AZD2014 was administrated were also irradiated with the same laser powers and resulted in no cell death. The same experiment was performed in HEK293 cells, where a similar result was observed (Supplementary Fig. S.5).

The cell death induced by photo-activation using 405 nm laser light was repeated using a femtosecond laser emitting at 800 nm with a power of 8.5 mW as the irradiation source. As before 7 μM of the drug and PI was administered and we observed the onset of cell death at 1.5 h post irradiation, with most of the cells undergoing cell death within 24 h (Supplementary Fig. S.5). Controls with no drug present but using the same laser power invoked no cell death. Experiments were performed on the spheroid model and similar results were observed (data not shown).

### 3.5 INK128 studies

A similar approach to the AZD2014 drug measurements described above was taken with another pan-mTOR inhibitor INK128. However, whilst INK128 has natural fluorescence properties and is taken up into living cells it is not as strong a fluorophore as AZD2014.

Rapamycin absorbs between 265 nm-315 nm whilst INK128 displayed absorbance below 330 nm with a peak at 293 nm (Supplementary Fig. S.6). The molecular extinction coefficients of rapamycin and INK128 were calculated as 41,425 M^−1^ cm^−1^ at 279 nm and 16,215 M^−1^ cm^−1^ at 293 nm respectively (Supplementary Fig. S.6). Rapamycin did not show any fluorescence emission (data not shown). Excitation of INK128 at 308 nm gave a broad emission, with a peak at 400 nm indicating that INK128 exhibits fluorescence properties (Supplementary Fig. S.6E). The corresponding emission spectrum, monitoring the intensity at 400 nm showed a peak of excitation at 308 nm.

The fluorescence lifetime of INK128 using FLIM was also measured using a concentration of 70 μM. Only weak fluorescence was observed. For multiphoton excitation using 600 nm a higher concentration of 3.2 mM was used, and gave a lifetime of 4.7 ± 0.13 ns in DMSO. The quantum yield of INK128 in DMSO was determined as 0.33 (+/− 0.03) using quinine sulphate as a standard (spectral data shown in (Supplementary Fig. S.6F) at 300 nm excitation. This value is lower than that obtained for AZD2014, indicating that INK128 is 30% less efficient at emitting light compared to AZD2014.

The localisation of INK128 in live HEK293 cells was also investigated in order to correlate any similarities to AZD2014. Initial attempts were made using one-photon excitation wavelengths, however, due to weak INK128 fluorescence emmsion, two-photon excitation was used to again minimise any unwanted cellular background signals. HEK293 cells were initially imaged over time using 600 nm excitation. As expected, the two-photon excitation gave low levels of autofluorescence at 0.9 mW (Supplementary Fig. S.7) from natural indole molecules such as tryptophan and derivatives observed below 380 nm [54]. HEK293 cells incubated with 70 μM of INK128 at room temperature were imaged over time with 600 nm excitation. Uptake of the molecule in cells (Supplementary Fig. S.7) at these detectable levels was observed and showed uptake within 1 min (k_uptake_ = 42 s) and a plateau within 2 min. Similar subcellular localisation of INK28 to that of AZD2014 was seen, with some fluorescence observed in the nucleus and greater accumulation of fluorescence in the cytosol with similar sub-structure localisation to that observed with AZD2014 (Supplementary Fig. S.7A). However, due to the low fluorescence emission and need for increasing concentrations deemed toxic to the cell to image the fluorescence of INK128, no further studies were performed.

## 4 Discussion

AZD2014 has been reported previously as naturally fluorescent, emitting in the 483 nm region [55]. The fluorimeter studies from our work support these findings and in addition we show that AZD2014 fluorescence (emission peak at 468 nm) can be obtained using either one-photon or two-photon excitation, the latter gave optimum results at 680 nm and between 730 and 850 nm. The solvent dependence properties of AZD2014 make it suitable to be used to detect and quantify its cellular uptake and cellular location in living cells in relation to studying directly its role as an mTOR inhibitor for treatment of cancer. However, as we observed AZD2014 interacting with several protein components that make up mTORC1, it is likely that AZD2014 simply interacts with several kinases but with varying affinities. This may also explain in part the non-specificity of AZD2014 towards both mTORC1 and mTORC2.

A key aspect of this work centres on the ability to directly image the mTOR inhibitor AZD2014 without additional modifications made. For example; previous studies have needed to conjugate rapamycin to external fluorophores such as nitrobenzoxadiazole (NBD) for imaging in killifish renal proximal tubules [39] as well as for *in-vitro* spectroscopy for studying binding of fluorescein-coupled rapamycin analogues to FKBP family proteins [38]. The addition of fluorophores to macrolide structures such as rapamycin and rapamycin analogues is unfavourable as the addition of a dye to a compound could hinder uptake, solubility, and also function [57]. The strong co-localisation of AZD2014 with fluorescently labelled Rheb GTPase (mTORC1 subunit protein) [14,58] known to tether mTORC1 to endomembrane structures such as the ER and Golgi [13], indicates that the drug naturally targets mTOR localisation sites. This is further supported by the finding that AZD2014 strongly colocalised with ER-Tracker and that FRET-FLIM showed direct physical interactions with mTORC1 proteins. The FLIM studies may indicate that AZD2014 could have a higher affinity (interaction efficiency) to S6K1 than the other mTORC1 proteins including Rheb. The implication of this is unknown and requires further investigation. The action and mechanism of AZD2014 may therefore have several aspects including targeting the individual proteins, particularly S6K1 as well as deregulating the mTOR signalling pathway completely.

The uptake of AZD2014 in real-time has been established in monolayer HEK293 cells and found to be taken up rapidly, within one minute and saturation reached within 10–15 min post administration. These findings are in line with previous work, for example using multiphoton excitation to monitor uptake of the anti-cancer drug combretastatin in mammalian cells (CHO) [59]. Here we also show that AZD2014 can penetrate multi-cellular HEK293 spheroids, reaching the core; the uptake rate in the outer surface was found to be in line with monolayers, with slower rates of uptake observed at larger depths. These findings are similar to experiments measuring drug uptake of combretastatin (*E*-CA4) in C8161 spheroids using confocal and multiphoton microscopy [60]. However, we highlight a more complex behaviour that cannot be fitted with a standard Michalis-Menten-like function: the outer layers show initial rapid accumulation followed by a reduction of fluorescence, suggesting exchange between different layers, while the inner core shows a biphasic fluorescence emission, with an increasing accumulation after the death of the outer cell shell. Here we emphasise the power of applying novel and emerging imaging techniques such as LSFM. The use of LSFM has allowed us to image thick large 3D samples faster than standard confocal microscopy and with lower average laser powers at the sample. The complex uptake characteristics determined in these studies allow for better understanding of the uptake process. For example, other studies have shown that spheroids exhibit altered mTOR signalling with a ~ 50% reduction in mTOR activity compared to 2D monolayers [61]. Understanding mTOR inhibitor uptake in a model that resembles as close to real tumours is imperative towards the understanding of mTOR activity in the tumour environment. Using immunofluorescence staining to probe for mTOR activity of downstream substrate phosphorylation, the recent study has shown a heterogeneous labelling (gradient effect), starting from the outer layers to the inner layers of fixed spheroids [61]. This highlights the significance for mTOR inhibitors to be designed to diffuse beyond the outer layers of tumours in a reasonable time. However, it is worth noting that this gradient staining showed lack of labelling in the core layers of the spheroid that may be an artefact from antibodies not being able to permeate through the spheroid layers [61]. Hence live cell imaging is imperative for these types of studies as well as for future drug screening.

Intriguingly, the treatment of spheroids with AZD2014 followed by 405 nm excitation led to the discovery of a novel photo-activated effect where the spheroid radius increased by 23% as measured by LSFM. This correlated with blebbing and cell swelling found in 2D monolayer cells and are signs of cell death [62]. The use of propidium iodide as a marker for necrosis (a specific type of cell death mechanism) to verify viability in both 2D monolayer studies and 3D cell spheroids showed light-activation of AZD2014 to induce cancer cell death. Light sheet microscopy provides superior imaging over conventional microscopy methods for thick samples (>50 μm) due to its ability to rapidly image live cell events in 3D and 4D over long time periods (several hours to days) with reduced photo-toxicity owing to low average laser power illumination [63]. Multiphoton microscopy (>600 nm excitation) using resonant scanners and digital light modulators also provides reduced cellular photo-toxicity at fast frame rates. However we did not compare this technique with our LSFM reported here. The light-induced activation of AZD2014 resulted in cells undergoing cell death using both 405 nm and 800 nm (two-photon) irradiation, a mechanism known to be specific to PDT [64]. Although the involvement of reactive oxygen species (ROS) is not always essential in inducing cell death [65] (Type IV mechanism), their relevance to AZD2014 has not been identified yet. Future work would entail co-imaging AZD2014 uptake with cell-permeant CellROX™ Deep Red reagent, a fluorogenic ROS sensor in order to ascertain if ROS are the immediate cause of cell death.

This work demonstrates a methodological approach to studying pharmacologically targeted inhibitors in living cells using multiple advanced light imaging techniques that may aid drug design, development and further the understanding of the mTOR pathway. In addition, utilizing the photosensitive nature of pan-mTOR inhibitors may provide a novel dual-mechanism for increasing the potency and efficacy against tumour growth and mTOR related disease states and indeed influencing other cellular functions of mTOR.

## 5 Conclusions

In summary, we have characterised the *in vitro* fluorescence characteristics of the pan-mTOR inhibitor AZD2014 and found it to be highly fluorescent with a quantum yield in DMSO = 0.47 ± 0.02 and in PBS = 0.11 ± 0.01. The inhibitor can be excited at both ~300 nm and ~ 400 nm permitting imaging by both single and multiphoton methods, the latter being particularly important to minimise excitation of native cellular components. AZD2014 is observed to be rapidly taken up by living cells with a half-life of ~1 min in both monolayer cells studied using confocal microscopy and 3D spheroids studied using laser light sheet imaging. The uptake into 3D multilayer spheroids shows complex uptake characteristics compared to 2D monolayer cell cultures. AZD2014 differentially exhibits a longer lifetime of 4.8 ± 0.5 ns in the cytoplasm compared to the nucleus 3.9 ± 0.4 ns in living cells, as obtained using FLIM. The inhibitor localises to the ER/Golgi network, a hub for mTOR activity. It was observed that AZD2014 directly interacts with the GFP-tagged mTORC1 proteins using FLIM. Upon light irradiation, AZD2014 causes cell death by necrosis in both 2D and 3D cell culture. The utilization of AZD2014 as both a pan-mTOR inhibitor and photosensitiser provides a novel strategy for combating cancer and tumour growth. The mTOR inhibitor INK128 was also found to be naturally fluorescent although with a significantly smaller extinction co-efficient which reduced its usefulness in long-term imaging studies. The use of excitation wavelengths of 400 nm of AZD2014 does present certain challenges for direct excitation within the cellular environment, however, in such cases multiphoton excitation can be achieved, for example using femtosecond pulsed 600 and 800 nm laser light. Both wavelengths show very good excitation processes of AZD2014 and 800 nm in particular giving a PDT effect.

## Funding

This research was funded by the 10.13039/501100000268BBSRC for an iCASE PhD studentship with Evotec Ltd. support (BB/L016052/1). We thank 10.13039/501100000271STFC for funding access to the 10.13039/100013266Central Laser Facility. Support from LaserLab Europe (EU2010 654148).

## Availability of data and materials

All raw data files including images will be available *via* the STFC ePubs hosting site for access by other researchers.

## Author's contribution

SWB, ARA, and AWP designed the research. AA, AC and SWB performed the imaging experiments and AA and AWP performed the spectroscopy experiments. AA generated all the plasmids. SRN and SDA prepared the 3D spheroid samples. All authors discussed and interpreted the results. AA and AC prepared the initial draft of the manuscript followed by all authors contributing to revisions.

## Declaration of Competing Interest

The authors declare that they have no known competing financial interests or personal relationships that could have appeared to influence the work reported in this paper.

## Appendix A Supplementary data

Supplementary material. Image 1
