# Supplementary material for: Directly imaging the localisation and photosensitization properties of the pan-mTOR inhibitor, AZD2014, in living cancer cells
Source: J Photochem Photobiol B. 2020 Dec;213:112055. doi: 10.1016/j.jphotobiol.2020.112055 (PMC7762844; doi:10.1016/j.jphotobiol.2020.112055)
Supplement: Supplementary file 1 — Supplementary material. [file mmc1.pptx]

## Slide 1
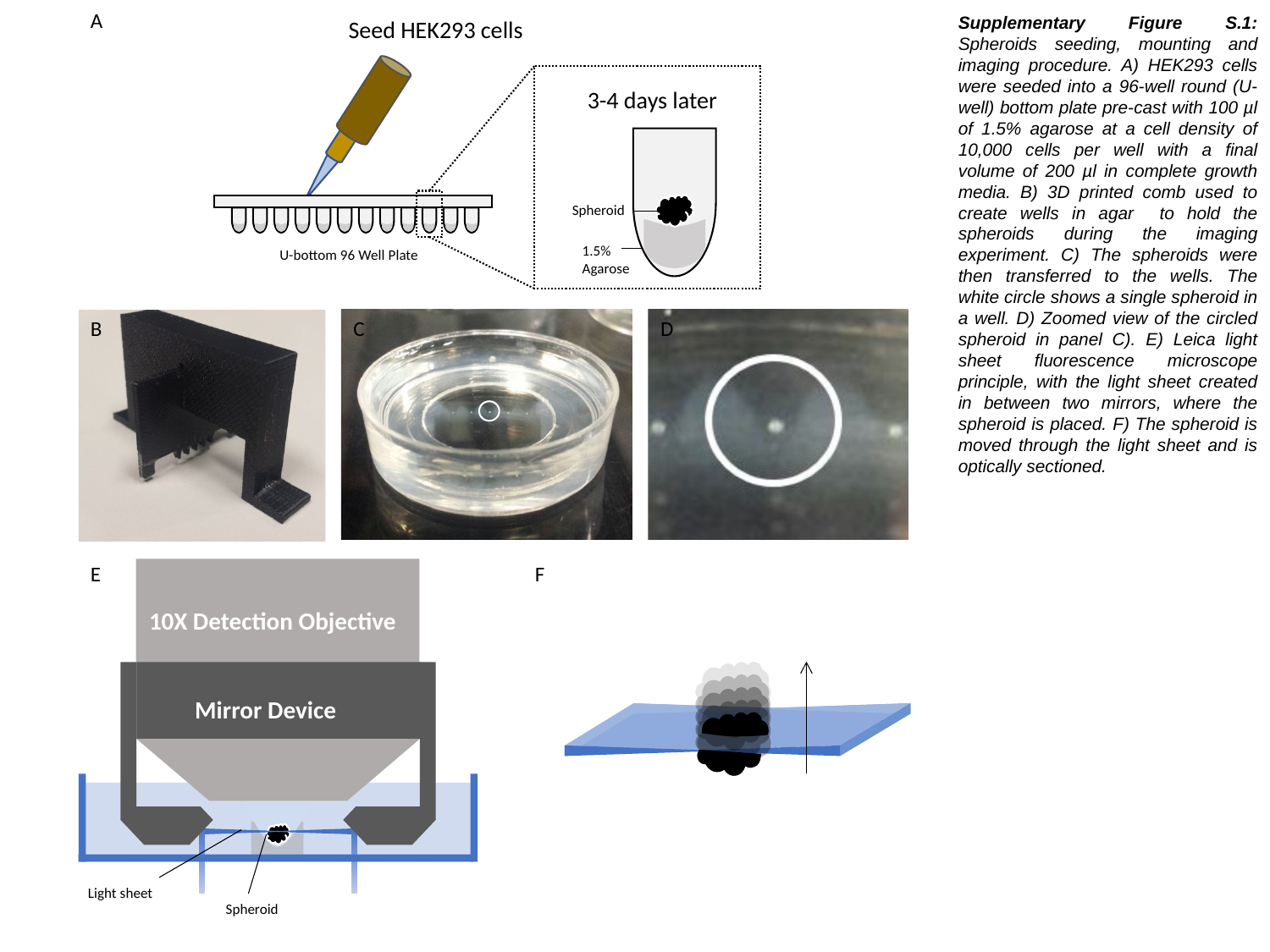

A
Supplementary Figure S.1: Spheroids seeding, mounting and imaging procedure. A) HEK293 cells were seeded into a 96-well round (U-well) bottom plate pre-cast with 100 µl of 1.5% agarose at a cell density of 10,000 cells per well with a final volume of 200 µl in complete growth media. B) 3D printed comb used to create wells in agar to hold the spheroids during the imaging experiment. C) The spheroids were then transferred to the wells. The white circle shows a single spheroid in a well. D) Zoomed view of the circled spheroid in panel C). E) Leica light sheet fluorescence microscope principle, with the light sheet created in between two mirrors, where the spheroid is placed. F) The spheroid is moved through the light sheet and is optically sectioned.
Seed HEK293 cells
3-4 days later
Spheroid
1.5% Agarose
U-bottom 96 Well Plate
B
C
D
E
F
10X Detection Objective
Mirror Device
Light sheet
Spheroid

## Slide 2
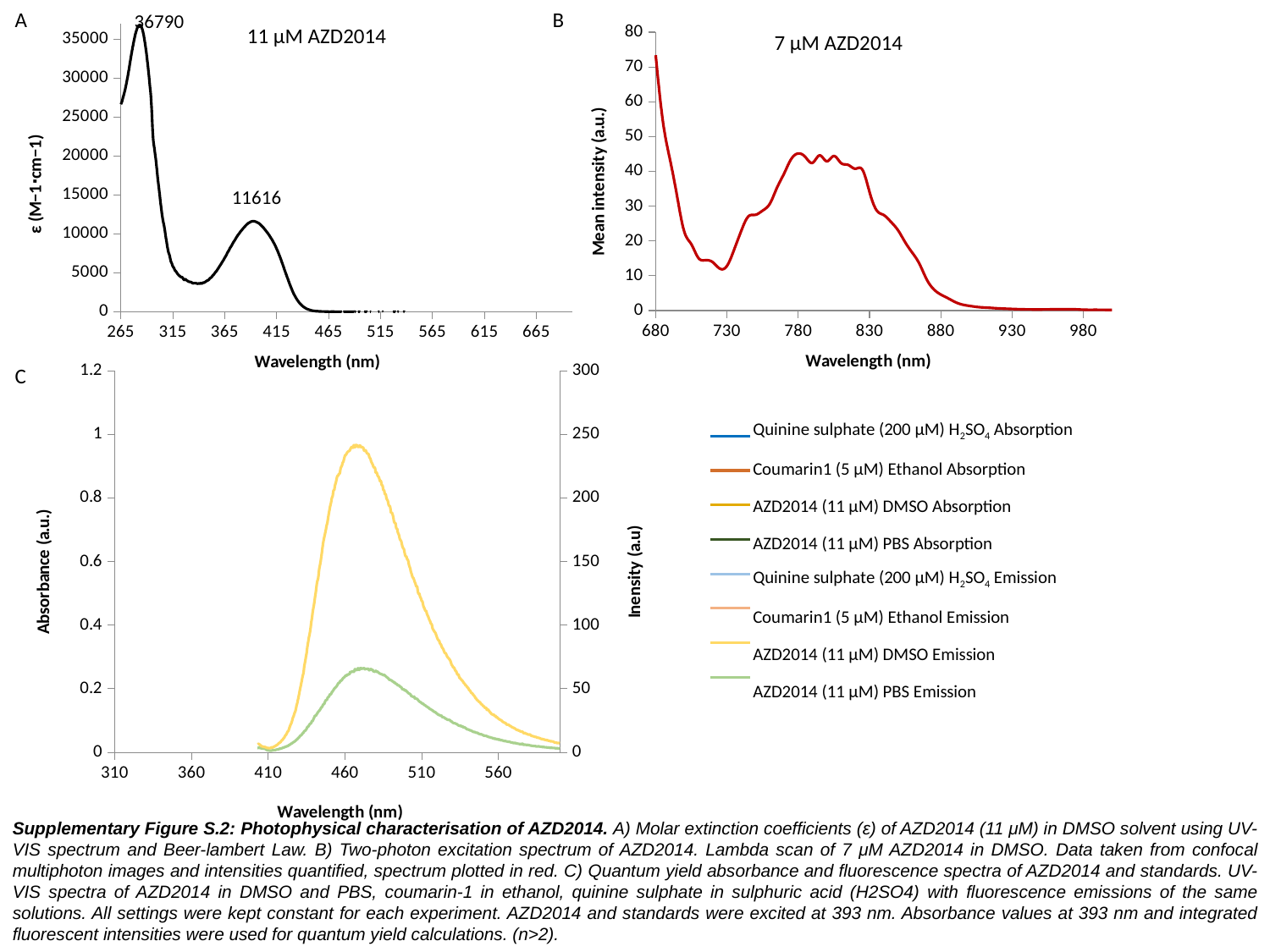

A
36790
### Chart
| Category | DMSO only |
|---|---|11616
11 µM AZD2014
B
### Chart
| Category | Intensity (a.u.) |
|---|---|7 µM AZD2014
C
### Chart
| Category | Quinine Sulphate (200 μM) H2SO4 Abs | Coumarin1 (5 μM) Ethanol Abs | AZD2014 (11 μM) DMSO Abs | AZD2014 (11 μM) PBS Abs | Quinine Sulphate (200 μM) H2SO4 Emission | Coumarin1 (5 μM) Ethanol Emission | AZD2014 (11 μM) DMSO Emission | AZD2014 (11 μM) PBS Emission |
|---|---|---|---|---|---|---|---|---|Quinine sulphate (200 µM) H2SO4 Absorption
Coumarin1 (5 µM) Ethanol Absorption
AZD2014 (11 µM) DMSO Absorption
AZD2014 (11 µM) PBS Absorption
Quinine sulphate (200 µM) H2SO4 Emission
Coumarin1 (5 µM) Ethanol Emission
AZD2014 (11 µM) DMSO Emission
AZD2014 (11 µM) PBS Emission
Supplementary Figure S.2: Photophysical characterisation of AZD2014. A) Molar extinction coefficients (ε) of AZD2014 (11 μM) in DMSO solvent using UV-VIS spectrum and Beer-lambert Law. B) Two-photon excitation spectrum of AZD2014. Lambda scan of 7 μM AZD2014 in DMSO. Data taken from confocal multiphoton images and intensities quantified, spectrum plotted in red. C) Quantum yield absorbance and fluorescence spectra of AZD2014 and standards. UV-VIS spectra of AZD2014 in DMSO and PBS, coumarin-1 in ethanol, quinine sulphate in sulphuric acid (H2SO4) with fluorescence emissions of the same solutions. All settings were kept constant for each experiment. AZD2014 and standards were excited at 393 nm. Absorbance values at 393 nm and integrated fluorescent intensities were used for quantum yield calculations. (n>2).

## Slide 3
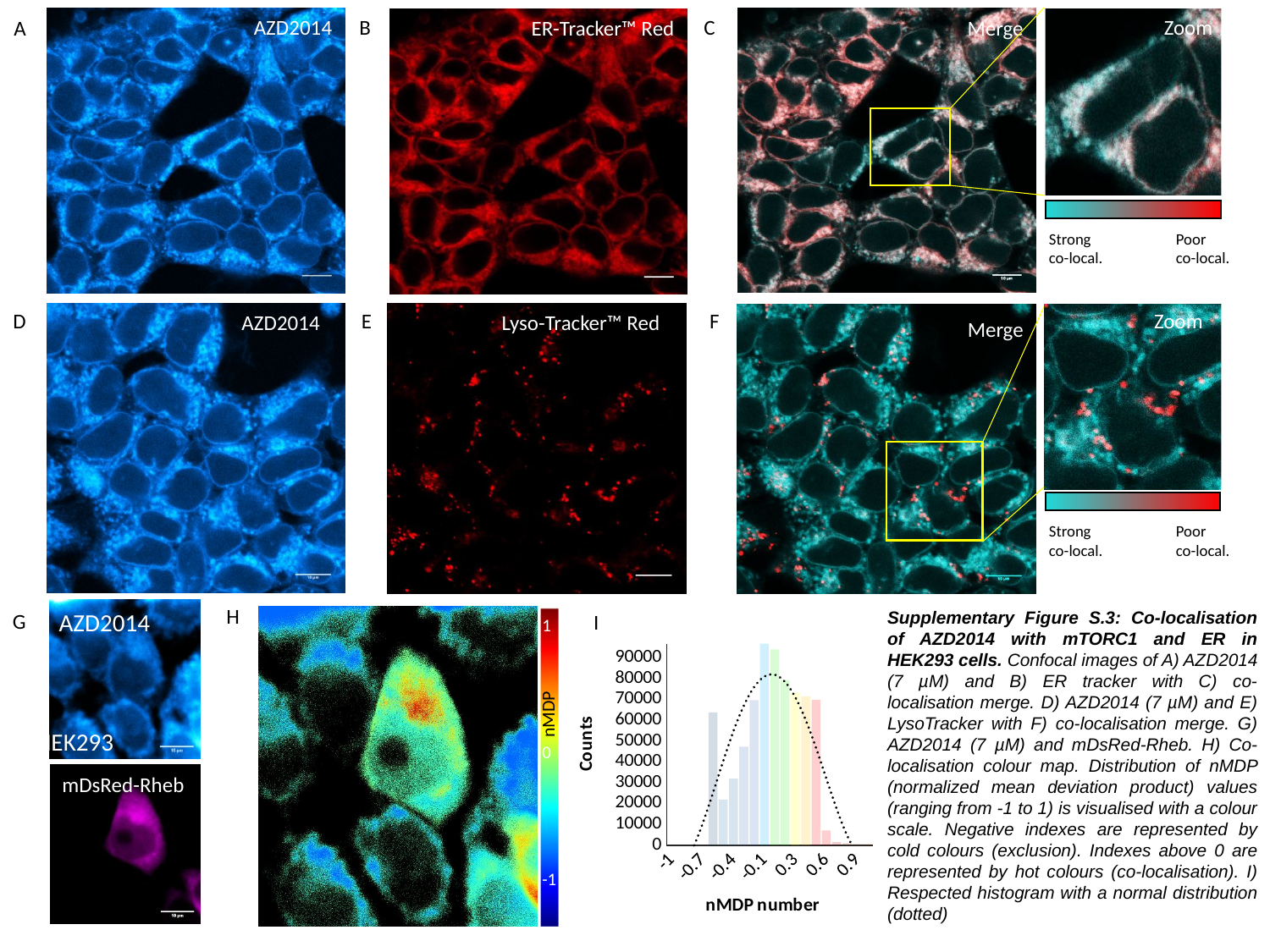

AZD2014
Merge
ER-Tracker™ Red
Lyso-Tracker™ Red
AZD2014
Merge
B
C
Zoom
A
Strong
co-local.
Poor
co-local.
E
F
Zoom
D
Strong
co-local.
Poor
co-local.
H
AZD2014
HEK293
Supplementary Figure S.3: Co-localisation of AZD2014 with mTORC1 and ER in HEK293 cells. Confocal images of A) AZD2014 (7 µM) and B) ER tracker with C) co-localisation merge. D) AZD2014 (7 µM) and E) LysoTracker with F) co-localisation merge. G) AZD2014 (7 µM) and mDsRed-Rheb. H) Co-localisation colour map. Distribution of nMDP (normalized mean deviation product) values (ranging from -1 to 1) is visualised with a colour scale. Negative indexes are represented by cold colours (exclusion). Indexes above 0 are represented by hot colours (co-localisation). I) Respected histogram with a normal distribution (dotted)
G
I
1
0
-1
nMDP
### Chart
| Category | Count |
|---|---|
| -1 | 2.0 |
| -0.9 | 4.0 |
| -0.8 | 31.0 |
| -0.7 | 117.0 |
| -0.6 | 63648.0 |
| -0.5 | 21949.0 |
| -0.4 | 31932.0 |
| -0.3 | 47227.0 |
| -0.2 | 69527.0 |
| -0.1 | 96636.0 |
| 0.1 | 93824.0 |
| 0.2 | 79146.0 |
| 0.3 | 73205.0 |
| 0.4 | 71300.0 |
| 0.5 | 69574.0 |
| 0.6 | 7139.0 |
| 0.7 | 1501.0 |
| 0.8 | 1039.0 |
| 0.9 | 646.0 |
| 1 | 670.0 |
mDsRed-Rheb

## Slide 4
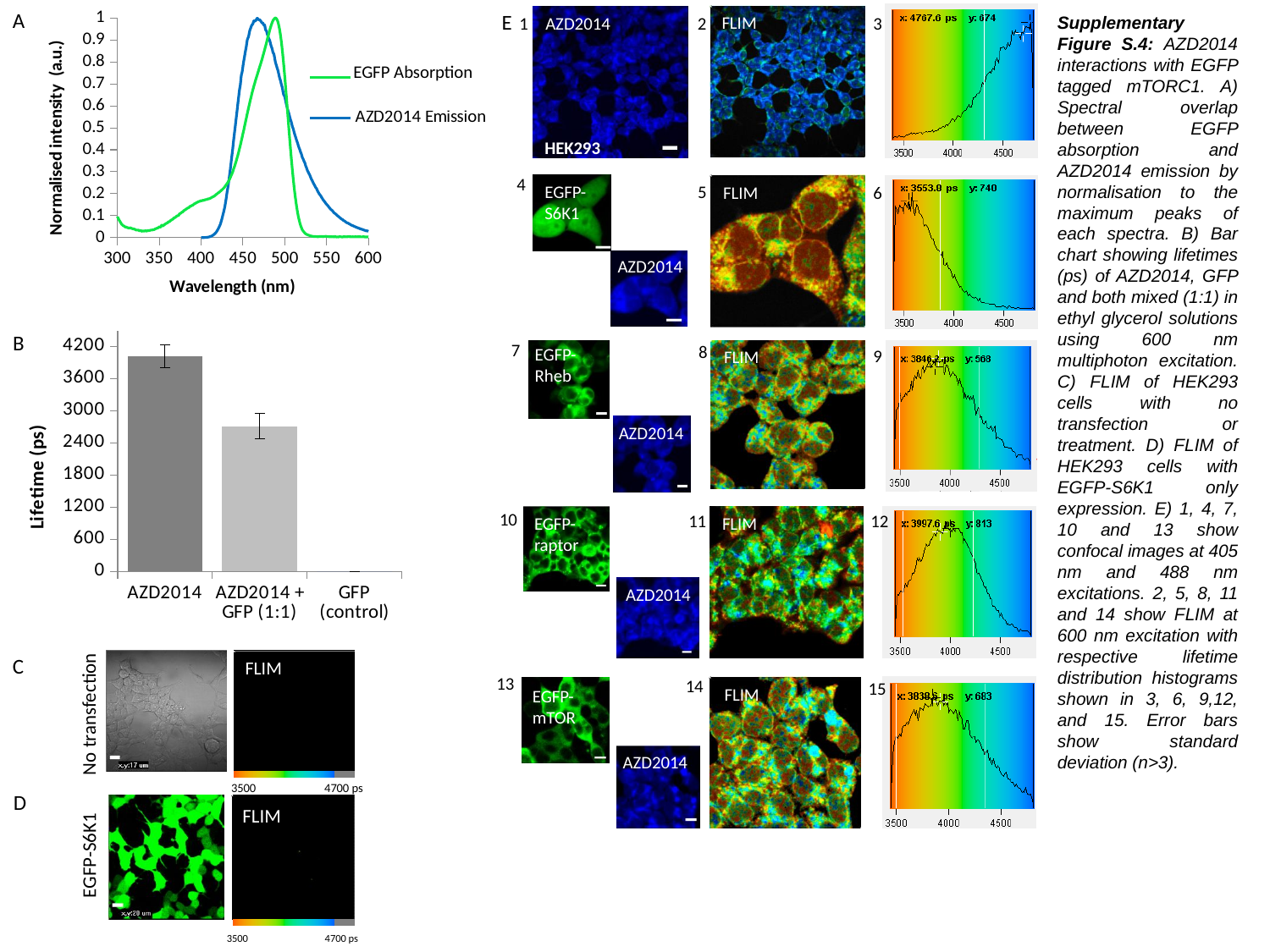

A
E
### Chart
| Category | | |
|---|---|---|
FLIM
2
AZD2014
3
1
HEK293
4
EGFP-S6K1
5
FLIM
6
AZD2014
8
EGFP-Rheb
9
FLIM
AZD2014
10
11
12
EGFP-raptor
FLIM
AZD2014
13
14
15
FLIM
EGFP-mTOR
AZD2014
Supplementary Figure S.4: AZD2014 interactions with EGFP tagged mTORC1. A) Spectral overlap between EGFP absorption and AZD2014 emission by normalisation to the maximum peaks of each spectra. B) Bar chart showing lifetimes (ps) of AZD2014, GFP and both mixed (1:1) in ethyl glycerol solutions using 600 nm multiphoton excitation. C) FLIM of HEK293 cells with no transfection or treatment. D) FLIM of HEK293 cells with EGFP-S6K1 only expression. E) 1, 4, 7, 10 and 13 show confocal images at 405 nm and 488 nm excitations. 2, 5, 8, 11 and 14 show FLIM at 600 nm excitation with respective lifetime distribution histograms shown in 3, 6, 9,12, and 15. Error bars show standard deviation (n>3).
EGFP Absorption
B
### Chart
| Category | |
|---|---|
| AZD2014 | 4019.6666666666665 |
| AZD2014 + GFP (1:1) | 2714.6 |
| GFP (control) | 0.0 |7
C
FLIM
3500 4700 ps
FLIM
EGFP-S6K1
3500 4700 ps
No transfection
D

## Slide 5
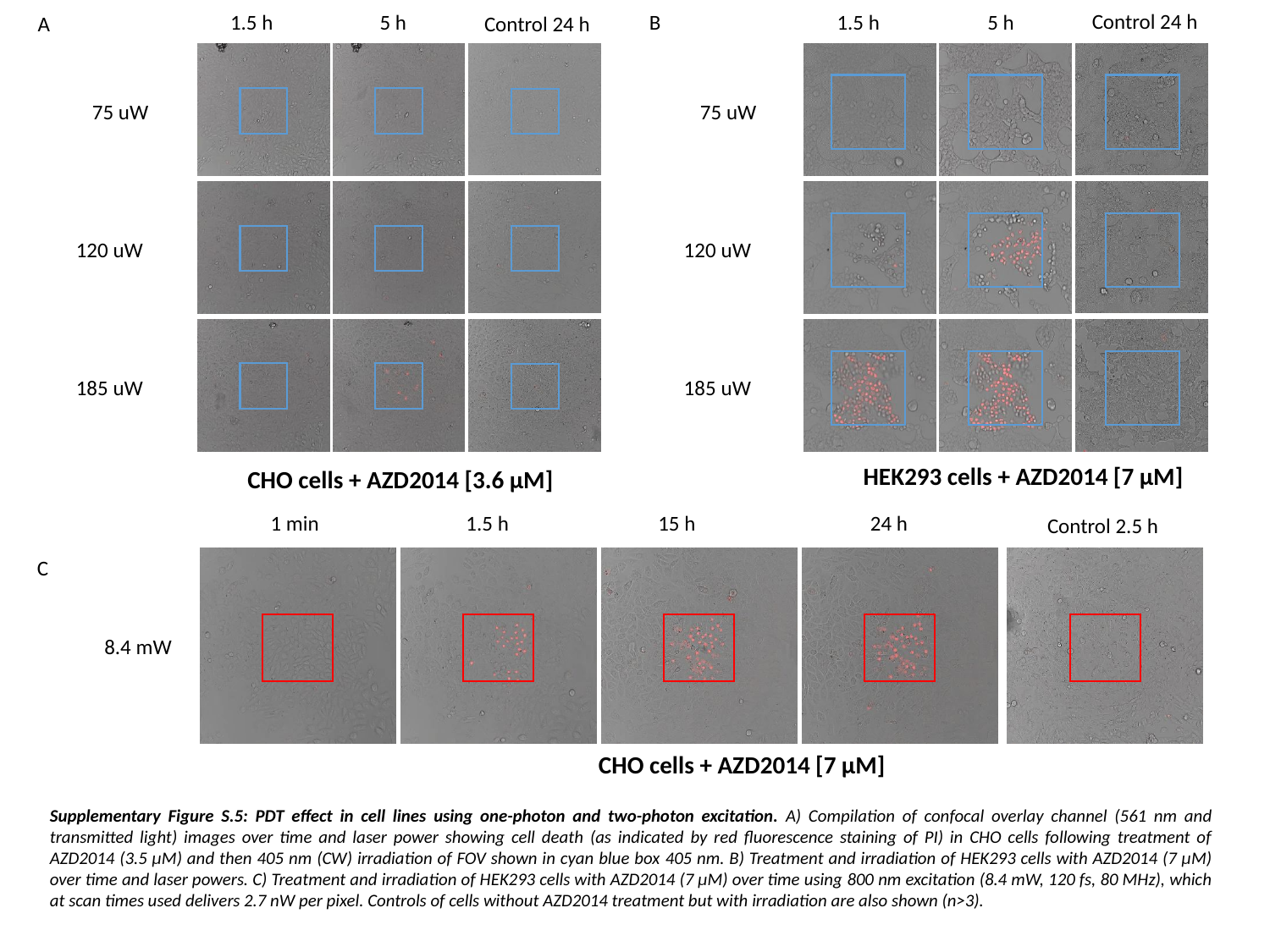

Control 24 h
1.5 h
5 h
B
1.5 h
5 h
A
Control 24 h
75 uW
75 uW
120 uW
120 uW
185 uW
185 uW
HEK293 cells + AZD2014 [7 µM]
CHO cells + AZD2014 [3.6 µM]
1 min
1.5 h
15 h
24 h
Control 2.5 h
C
8.4 mW
CHO cells + AZD2014 [7 µM]
Supplementary Figure S.5: PDT effect in cell lines using one-photon and two-photon excitation. A) Compilation of confocal overlay channel (561 nm and transmitted light) images over time and laser power showing cell death (as indicated by red fluorescence staining of PI) in CHO cells following treatment of AZD2014 (3.5 µM) and then 405 nm (CW) irradiation of FOV shown in cyan blue box 405 nm. B) Treatment and irradiation of HEK293 cells with AZD2014 (7 µM) over time and laser powers. C) Treatment and irradiation of HEK293 cells with AZD2014 (7 µM) over time using 800 nm excitation (8.4 mW, 120 fs, 80 MHz), which at scan times used delivers 2.7 nW per pixel. Controls of cells without AZD2014 treatment but with irradiation are also shown (n>3).

## Slide 6
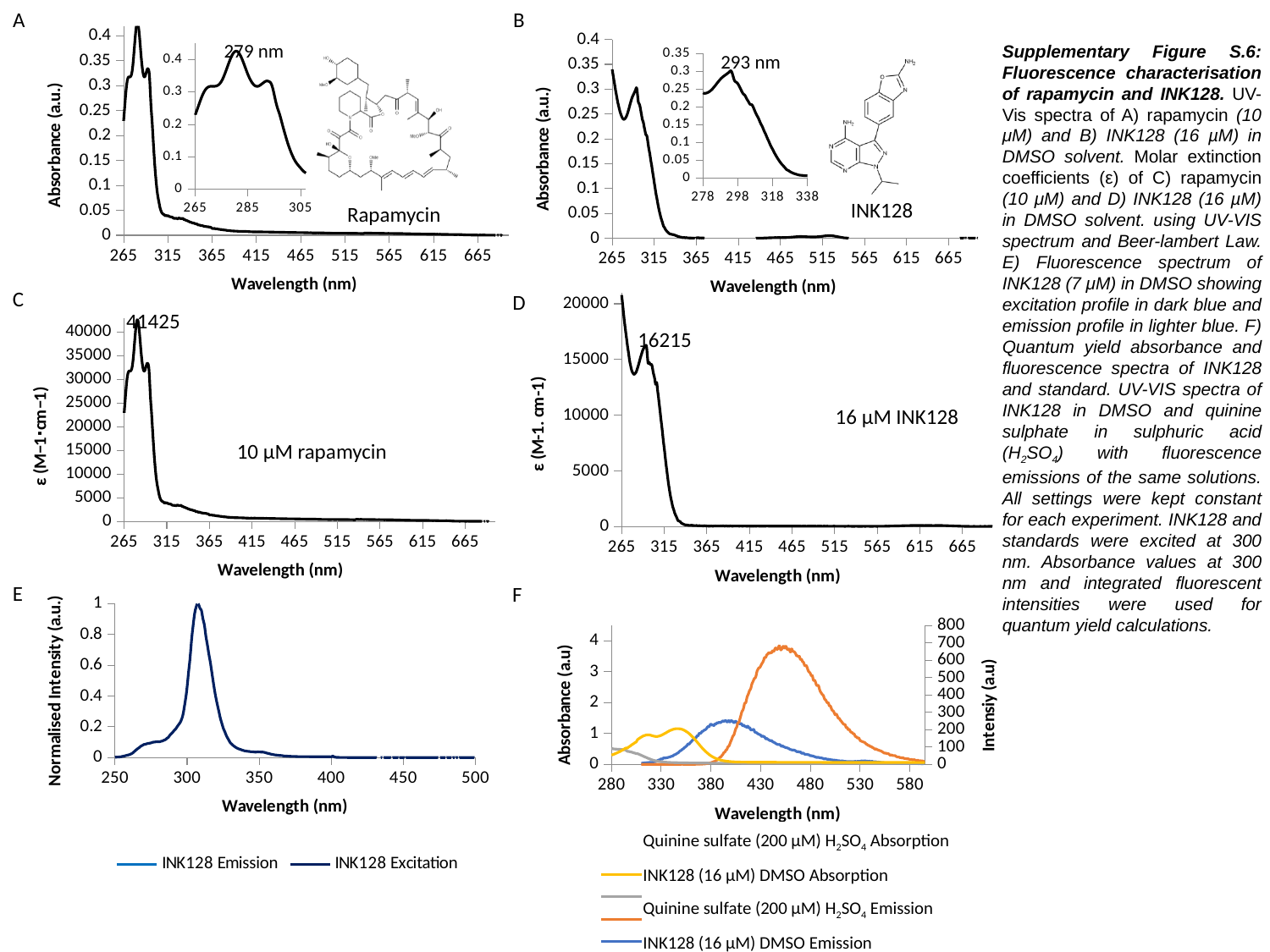

A
B
### Chart
| Category | |
|---|---|
### Chart
| Category | INK128 with DMSO |
|---|---|279 nm
Supplementary Figure S.6: Fluorescence characterisation of rapamycin and INK128. UV-Vis spectra of A) rapamycin (10 μM) and B) INK128 (16 µM) in DMSO solvent. Molar extinction coefficients (ε) of C) rapamycin (10 μM) and D) INK128 (16 µM) in DMSO solvent. using UV-VIS spectrum and Beer-lambert Law. E) Fluorescence spectrum of INK128 (7 μM) in DMSO showing excitation profile in dark blue and emission profile in lighter blue. F) Quantum yield absorbance and fluorescence spectra of INK128 and standard. UV-VIS spectra of INK128 in DMSO and quinine sulphate in sulphuric acid (H2SO4) with fluorescence emissions of the same solutions. All settings were kept constant for each experiment. INK128 and standards were excited at 300 nm. Absorbance values at 300 nm and integrated fluorescent intensities were used for quantum yield calculations.
### Chart
| Category | |
|---|---|
### Chart
| Category | INK128 with DMSO |
|---|---|293 nm
Rapamycin
INK128
C
D
41425
### Chart
| Category | DMSO only |
|---|---|16215
16 µM INK128
10 µM rapamycin
### Chart
| Category | |
|---|---|E
### Chart
| Category | INK128 Emission | INK128 Excitation |
|---|---|---|F
### Chart
| Category | Quinine sulfate (200 µM) H2SO4 Abs | INK128 (16 µM) DMSO Abs | Quinine sulfate (200 µM) H2SO4 Emission | INK128 (16 µM) DMSO Emission |
|---|---|---|---|---|Quinine sulfate (200 µM) H2SO4 Absorption
INK128 (16 µM) DMSO Absorption
Quinine sulfate (200 µM) H2SO4 Emission
INK128 (16 µM) DMSO Emission

## Slide 7
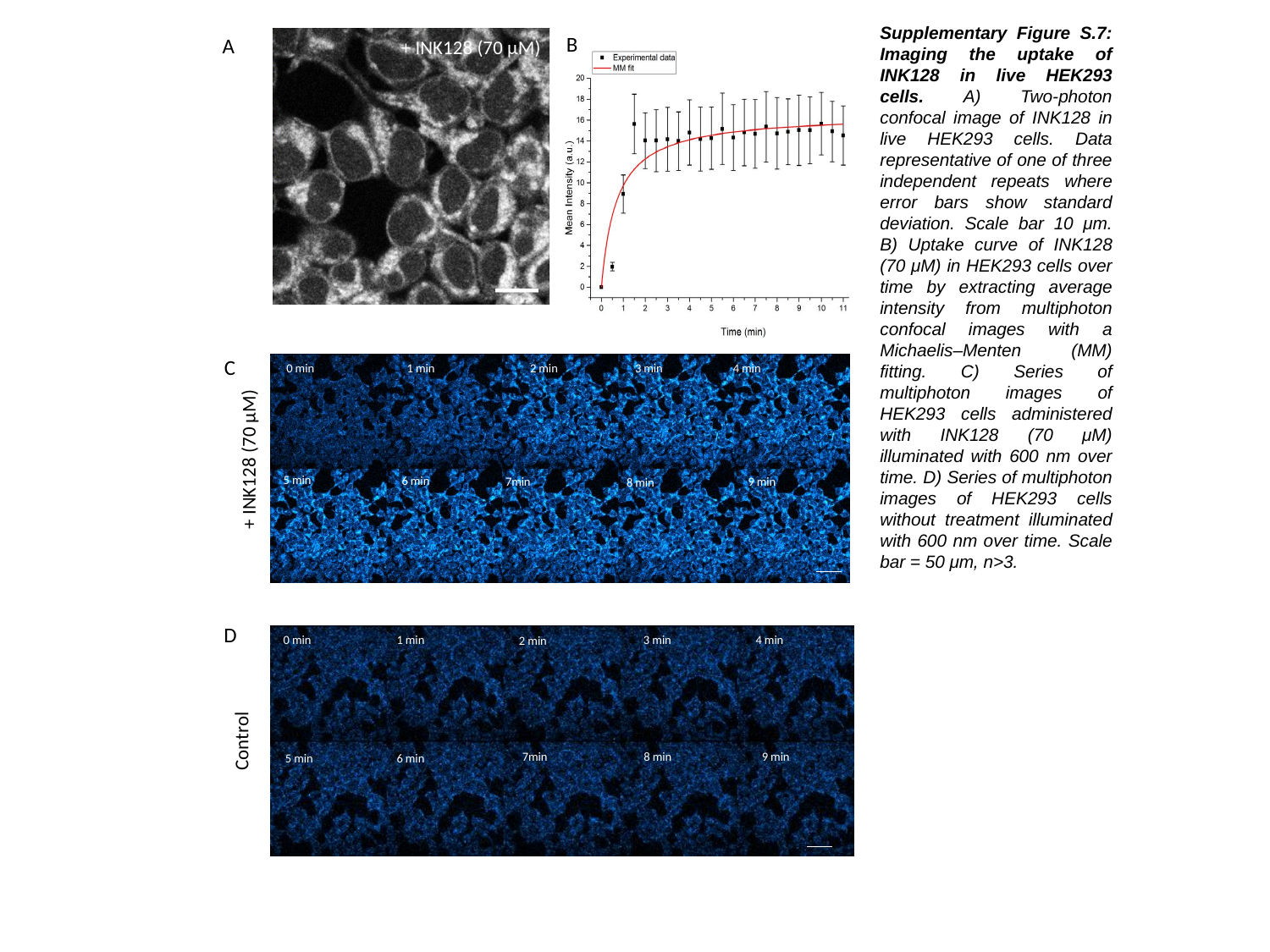

Supplementary Figure S.7: Imaging the uptake of INK128 in live HEK293 cells. A) Two-photon confocal image of INK128 in live HEK293 cells. Data representative of one of three independent repeats where error bars show standard deviation. Scale bar 10 μm. B) Uptake curve of INK128 (70 μM) in HEK293 cells over time by extracting average intensity from multiphoton confocal images with a Michaelis–Menten (MM) fitting. C) Series of multiphoton images of HEK293 cells administered with INK128 (70 μM) illuminated with 600 nm over time. D) Series of multiphoton images of HEK293 cells without treatment illuminated with 600 nm over time. Scale bar = 50 μm, n>3.
B
A
+ INK128 (70 µM)
C
1 min
0 min
3 min
4 min
2 min
+ INK128 (70 µM)
5 min
6 min
9 min
7min
8 min
D
1 min
3 min
0 min
4 min
2 min
Control
8 min
9 min
7min
5 min
6 min
